# Distinct Red Blotch Disease Epidemiological Dynamics in Two Nearby Vineyards

**DOI:** 10.3390/v15051184

**Published:** 2023-05-17

**Authors:** Madison T. Flasco, Elizabeth J. Cieniewicz, Sarah J. Pethybridge, Marc F. Fuchs

**Affiliations:** 1 School of Integrative Plant Science, Plant Pathology and Plant-Microbe Biology Section, Cornell University, Geneva, NY 14456, USA; sjp277@cornell.edu (S.J.P.); marc.fuchs@cornell.edu (M.F.F.); 2 Department of Plant and Environmental Sciences, Clemson University, Clemson, SC 29634, USA; ecienie@clemson.edu

**Keywords:** grapevine red blotch virus, *Grablovirus*, *Geminiviridae*, *Vitis vinifera*, *Spissistilus festinus*, spatial analysis, epidemiology

## Abstract

Grapevine red blotch virus (GRBV) causes red blotch disease and is transmitted by the three-cornered alfalfa hopper, *Spissistilus festinus*. GRBV isolates belong to a minor phylogenetic clade 1 and a predominant clade 2. Spatiotemporal disease dynamics were monitored in a 1-hectare ‘Merlot’ vineyard planted in California in 2015. Annual surveys first revealed disease onset in 2018 and a 1.6% disease incidence in 2022. Ordinary runs and phylogenetic analyses documented significant aggregation of vines infected with GRBV clade 1 isolates in one corner of the vineyard (*Z* = −4.99), despite being surrounded by clade 2 isolates. This aggregation of vines harboring isolates from a non-prevalent clade is likely due to infected rootstock material at planting. GRBV clade 1 isolates were predominant in 2018–2019 but displaced by clade 2 isolates in 2021–2022, suggesting an influx of the latter isolates from outside sources. This study is the first report of red blotch disease progress immediately after vineyard establishment. A nearby 1.5-hectare ‘Cabernet Sauvignon’ vineyard planted in 2008 with clone 4 (CS4) and 169 (CS169) vines was also surveyed. Most CS4 vines that exhibited disease symptoms one-year post-planting, likely due to infected scion material, were aggregated (*Z* = −1.73). GRBV isolates of both clades were found in the CS4 vines. Disease incidence was only 1.4% in non-infected CS169 vines in 2022 with sporadic infections of isolates from both clades occurring via secondary spread. Through disentangling GRBV infections due to the planting material and *S. festinus*-mediated transmission, this study illustrated how the primary virus source influences epidemiological dynamics of red blotch disease.

## 1. Introduction

Red blotch disease was first described in 2008 in *Vitis vinifera* ‘Cabernet Sauvignon’ in California [1]. In the early 2010s, grapevine red blotch virus (GRBV) was found to be associated with diseased vines [2,3] and later described as the causative agent of the disease [4]. Infected red grape cultivars exhibit foliar reddening, while infected white grape cultivars experience foliar chlorosis and necrosis [5,6,7]. Diseased vines also exhibit delayed fruit ripening, reduced fruit quality and yield, and ultimately deleterious effects on wine composition [5,7,8,9,10]. These symptoms can result in economic losses reported to range from $2200 to $68,500 per hectare over the course of the productive lifespan of a vineyard in the United States [11].

GRBV is graft-transmissible [6,12] and the only known natural hosts of GRBV are *Vitis* spp. [12,13,14]. These include grape cultivars and rootstock genotypes [6,12], as well as free-living vines in Northern California [15,16,17] and Southern Oregon [18]. 

GRBV is a member of the species of *Grapevine red blotch virus* in the genus *Grablovirus* in the family *Geminiviridae* [19,20]. The viral genome is composed of a single stranded DNA molecule encompassing seven bidirectional open reading frames (ORFs) [12,21]. GRBV isolates belong to two distinct phylogenetic clades: GRBV clade 1 contains fewer isolates with up to 94.8% sequence identity, and GRBV clade 2 contains most isolates with 98.8% or higher sequence identity. Inter-clade genetic variability indicates up to 8.5% sequence divergence [22]. GRBV isolates from both clades are involved in disease etiology, though no biological differences between the clades are known [4].

GRBV is detected in vineyards across the United States [22,23] and throughout North America [24,25,26,27]. The virus has also been reported in multiple countries around the world, including South Korea [28], Switzerland [29], Argentina [30], India [31], Italy [32], France [33], and Australia [34]. This widespread distribution of GRBV is attributed to the dissemination of infected planting material [2,35].

Virus secondary spread has been reported in northern California [5,16,36] and southern Oregon [18,37]. Spatial analysis via distance indices (SADIE) and ordinary runs analyses indicated aggregations of diseased vines in both geographical regions. The association function of SADIE determined that spatial patterns of virus incidence were associated with the previous year’s incidence in both the Oregon and California studies [18,37,38]. Furthermore, the aggregation patterns of infected vines combined with randomly isolated symptomatic vines implicated a flying hemipteran vector in GRBV spread [18,37,38]. This observation in conjunction with insect surveys in diseased vineyards led to the identification of the three-cornered alfalfa hopper, *Spissistilus festinus*, as a vector candidate [5,16,36]. Subsequently, *S. festinus* was shown to transmit GRBV in greenhouse settings [13,39,40] and the vineyard [41], solidifying the insect’s epidemiological role in GRBV spread. Isolates from both GRBV phylogenetic clades are transmitted by *S. festinus* in the greenhouse [39] and vineyard [41].

All previous epidemiological studies of GRBV were performed in diseased vineyards starting 6–16 years post-planting [5,16,18,36,37], with the exception of one vineyard in southern Oregon where entire rows of grapevines were interplanted in an already established block five years post-planting, and surveyed five years later, i.e., ten years after the original planting [18]. It should be noted that three vineyards were surveyed three years post-planting, though these surveys were only conducted in a single year and changes in disease incidence could not be analyzed [18]. None of these studies monitored the onset of red blotch disease immediately after the establishment of a vineyard block. Similarly, no epidemiological study centered on the involvement of GRBV isolates from phylogenetic clades 1 or 2 in disease spread. In addition, despite great strides in understanding disease epidemiology, no information is available on the onset of red blotch disease symptoms resulting from infected planting material versus *S. festinus*-mediated spread of GRBV. Of interest, the occurrence of secondary spread from local (within vineyard) and background (outside the vineyard of interest) sources was suspected [36].

The main objectives of this study were to: (i) characterize disease onset and spread in a vineyard beginning at planting, (ii) determine the implication of distinct GRBV isolates in the spatiotemporal patterns of diseased vines, and (iii) disentangle infections resulting from the planting material and *S. festinus*-mediated transmission of GRBV in a ‘Merlot’ vineyard block. We also monitored disease spread in a nearby ‘Cabernet Sauvignon’ vineyard block with a large established area of inoculum to validate observations made in the ‘Merlot’ block. We hypothesized that most disease outbreaks are due to infected planting material, and that the timing of disease onset differs based on the primary source of GRBV inoculum from the scion or rootstock.

## 2. Materials and Methods

### 2.1. Study Vineyard Selection

**Figure 1:**
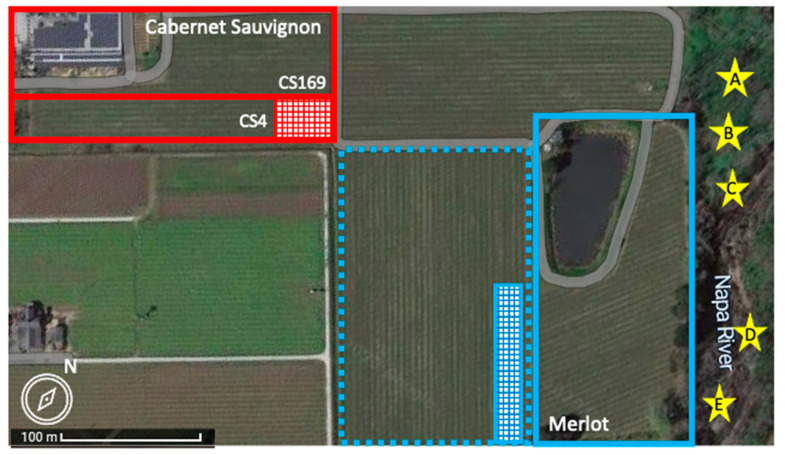
Satellite image of two vineyards surveyed in this study for grapevine red blotch virus (GRBV). Outlined in blue is a ‘Merlot’ vineyard block planted in 2015. West of ‘Merlot’ block of interest is the other ‘Merlot’ block (outlined in dotted blue) exhibiting foliar reddening for which leaf samples were collected from 20 randomly selected symptomatic vines (blue crosshatched box) to determine GRBV phylogenetic clade distribution. Outlined in red is a ‘Cabernet Sauvignon’ vineyard planted with clones 4 (CS4) and 169 (CS169) in 2008. CS4 vines were planted along the southern portion of this block (outlined in red). Sample collections of 189 CS4 symptomatic vines symptomatic (red crosshatched box) were conducted to determine GRBV clade distribution. East of the ‘Cabernet Sauvignon’ and north of the ‘Merlot’ blocks is a GRBV-infected ‘Cabernet franc’ block [5,17,36,38]. East of all these blocks is a riparian area with the Napa River. Yellow stars indicate location of free-living vines (A–E) sampled for GRBV in riparian area. Adapted with permission from Google Imagery ©2023 NES/Airbus, Maxar Technologies, USDA/FPAC/GEO, Map data ©2023.

Two neighboring vineyards in Napa County, California with vines showing red blotch disease symptoms and GRBV presence confirmed via PCR were selected for this study (Figure 1). A 1-hectare *V. vinifera* ‘Merlot’ clone 181 grafted on rootstock 101–14 Mgt planted in 2015 was selected due to the opportunity to monitor disease incidence from planting and its proximity (10 m) to a riparian area (Figure 1). A 1.5- hectare *V. vinifera* ‘Cabernet Sauvignon’ vineyard was chosen for this study based on the previous epidemiological observations of the block [36]. The block was planted in 2008 using vines of clones 4 (CS4) and 169 (CS169) grafted onto rootstock 101–14 Mgt on the southern and northern ends of the block, respectively. Approximately 60% of the block consists of CS4 plantings while the remaining area is CS169 (Figure 1). This material was sourced from two different nurseries. Further interest in this vineyard block resulted from GRBV presence in almost all CS4 vines one year after planting [36] and its distance (260 m) from a riparian area (Figure 1). 

Both vineyard blocks selected for this study were adjacent to a heavily infected ‘Cabernet franc’ vineyard that has been continuously surveyed for GRBV presence in individual vines since 2014; the infected vineyard lies east of the ‘Cabernet Sauvignon’ block of interest and north of the ‘Merlot’ block of interest (Figure 1). Another heavily diseased ‘Merlot’ block that lies west of the ‘Merlot’ block of interest, which is referred to as the other ‘Merlot’ block, was selected to characterize the GRBV clade present in surrounding inoculum sources (Figure 1).

All vineyard blocks selected for this study had a vertical shoot position trellis system. Similarly, in all blocks, vines were spaced 1.2 and 2.1 m within and between rows, respectively. Conventional irrigation and pest management practices for vineyards in Napa County were applied to our study blocks throughout the duration of this work.

### 2.2. Survey for GRBV Incidence in the Two Study Vineyards

Every vine in the ‘Merlot’ vineyard of interest was visually surveyed for typical red botch disease symptoms, i.e., foliar red blotches, in October during the 2015–2019, 2021, and 2022 growing seasons. No surveys were performed in 2020 due to travel restrictions with regard to the COVID-19 pandemic. Six leaves and petioles were collected from the base of the vine canopy close to the trunk (three leaves and petioles from each cordon) in newly symptomatic vines each year for confirmation of GRBV presence via PCR, utilizing petiole tissue and GRBV genotype analyses.

Every CS169 vine in the ‘Cabernet Sauvignon’ vineyard was similarly monitored for disease symptoms in 2017 and 2018 [36], and again in 2019, 2021, and 2022 (this study). No surveys were performed in 2020 due to travel restrictions related to the COVID-19 pandemic. Similar to the ‘Merlot’ vineyard, leaves and petioles from newly symptomatic vines in the CS169 plantings were collected for GRBV testing and isolate genotyping. In addition, leaves and petioles from 189 symptomatic CS4 vines were collected to assess the ratio of GRBV clade 1 and GRBV clade 2 isolates in the section of the vineyard with high disease incidence (Figure 1). 

### 2.3. Survey for GRBV Incidence Surrounding the Vineyard Blocks of Interest

To better understand GRBV incidence in vineyards surrounding the ‘Merlot’ and ‘Cabernet Sauvignon’ vineyards of interest, leaf tissue was collected from 20 selected vines that exhibited characteristic foliar reddening of red blotch disease in the other ‘Merlot‘ block, which was adjacent (on the west side) to the ‘Merlot’ block of interest (Figure 1), to assess the presence of GRBV and clade 1 or clade 2 isolates. 

Adjacent to the ‘Merlot’ block of interest on the opposite side (east of the block) is a riparian area densely populated with free-living grapevines. Leaf tissue from free-living vines was collected a minimum of 30 m apart to ensure collection from different independent grapevines (Figure 1). 

### 2.4. Nucleic Acid Extraction from Grapevine Tissue and GRBV Detection via PCR

Genomic DNA was isolated from grapevine leaf material using the H.P. Plant DNA Mini Kit (Omega Bio-Tek, Norcross, GA, USA) or the MagMAX-96 Al/ND Isolation Kit (Thermo Fisher Scientific, Waltham, MA, USA) on a KingFisher instrument (Thermo Fisher Scientific, Waltham, MA, USA). 

GRBV presence was determined in nucleic acid extracts via multiplex PCR using primer pairs hybridizing to the coat protein and replication ORFs of the viral genome [22,36,38]. DNA amplicons were analyzed via gel electrophoresis and visualized using UV illumination post-staining with GelRed (Biotium, Fremont, CA, USA).

### 2.5. GRBV Phylogenetic Clade Identification

To determine the phylogenetic clade of GRBV isolates in symptomatic, infected grapevines, PCR products corresponding to a fragment of the GRBV replication ORF [22,36,38] were Sanger sequenced at the Cornell Biotechnology Resource Center in Ithaca, New York. GRBV sequences were assembled and analyzed using DNASTAR Lasergene software suite, version 14.1. Additionally, some PCR products underwent restriction digestion with AleI-v2 (New England Biolabs, Ipswich, MA, USA) to determine the GRBV phylogenetic clade, as previously described [41]. Digestions were resolved via electrophoresis on agarose gels and visualized using UV illumination post-staining with GelRed (Biotium, Fremont, CA, USA). Restriction digests of approximately 201 and 117 bp in size reflected a GRBV clade 1 isolate, while a single uncut 318 bp fragment indicated a GRBV clade 2 isolate [41].

### 2.6. Spatial Analysis of Symptomatic Grapevines in the ‘Merlot’ Block of Interest

Ordinary runs analysis was used to determine if the spatial pattern of symptomatic vines in a single dimension was aggregated or random [42,43]. Each “run” was defined as one or more vines of the same status (healthy versus diseased). The analysis was conducted on a sub-portion of the ‘Merlot’ block (rows 1–24) on individual rows, when disease incidence was greater than 5%. Such analyses were also performed on the ‘Merlot’ block (rows 1–24) and a sub-portion of the CS4 plantings (Figure 1) as one contiguous row. The null hypothesis assumed a random distribution of diseased vines. Areas of the vineyard block could be considered non-random or aggregated when the *Z*-statistic was Z≤−1.64 (p≤0.05), meaning that the observed number of runs was significantly different from the expected number under the null hypothesis [42,43]. 

### 2.7. Genotyping Free-Living Grapevines

Ten mature leaves were collected from each free-living vine (Figure 1) to assess their ancestry. Genotype analysis was performed as described previously [16,17,40,44] using eight simple-sequence repeat (SSR) markers [45,46,47,48]. At each locus, alleles were assigned to *V. californica* or a different *Vitis* spp. 

## 3. Results

### 3.1. Onset of Red Blotch Disease Symptoms in a ‘Merlot’ Block 

**Figure 2:**
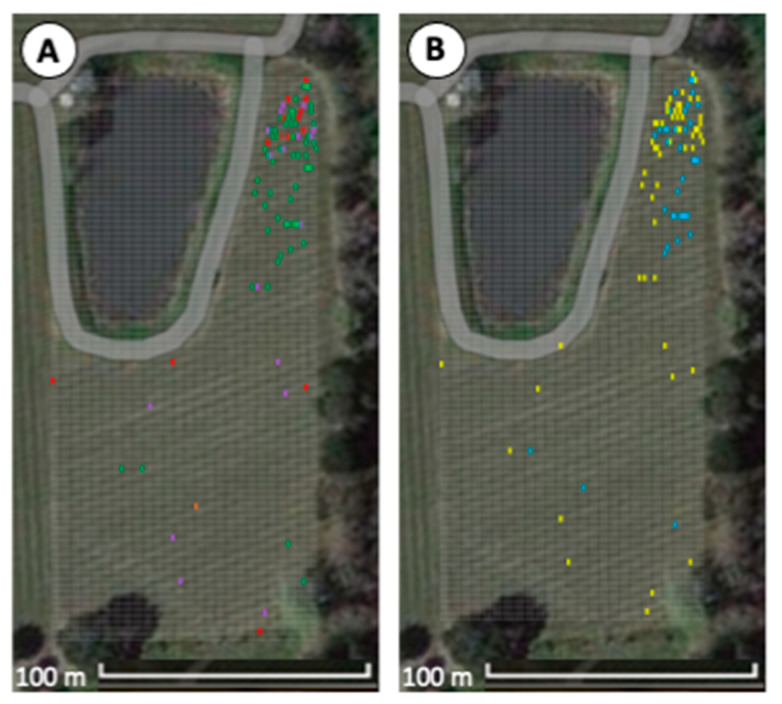
Grapevine red blotch virus (GRBV) infected vines in ‘Merlot’ vineyard block in 2015–2022. (**A**) Symptomatic grapevines observed in 2018 (red), 2019 (orange), 2021 (purple), and 2022 (green) are indicated. GRBV presence was confirmed in symptomatic vines via PCR. (**B**) Phylogenetic clade distribution in GRBV-infected vines with clade 1 isolates are depicted in blue, and clade 2 isolates are shown in yellow. A pond is visible on the upper left corner of the satellite image. Adapted with permission from Google Imagery ©2023 NES/Airbus, Maxar Technologies, USDA/FPAC/GEO, Map data ©2023.

The ‘Merlot’ block was planted in 2015, and red blotch disease symptoms were first observed in 2018 in only 14 grapevines (0.2%, 14/5731) (Figure 2A). Notably, 10 of the 14 newly symptomatic vines were in the northern corner of the ‘Merlot’ block (rows 1–24), whereas the remaining four were dispersed south of this grouping. In 2019, only one vine was newly symptomatic and found in the middle of the vineyard block (0.2%, 15/5731). In 2021, 17 newly symptomatic vines were identified for a total of 32 symptomatic vines (0.06%, 32/5731). Ten of the new infections were again located in the northern corner of the ‘Merlot’ block, while the remaining seven were randomly dispersed (Figure 2A). In 2022, 58 newly infected vines were identified (1.6%, 90/5731). Together, the number of newly symptomatic grapevines increased annually in the ‘Merlot’ block from 0.2% in 2018 to 1.6% in 2022, and the overall disease incidence remained low (Supplemental Table S1). Notably, most (74%, 67/90) newly symptomatic grapevines were concentrated in the northern corner of the ‘Merlot’ block exhibiting high disease incidence (Figure 2A). 

### 3.2. Spatial Analyses Confirm Aggregation of Diseased Grapevines at One Corner of the ‘Merlot’ Block 

**Figure 3:**
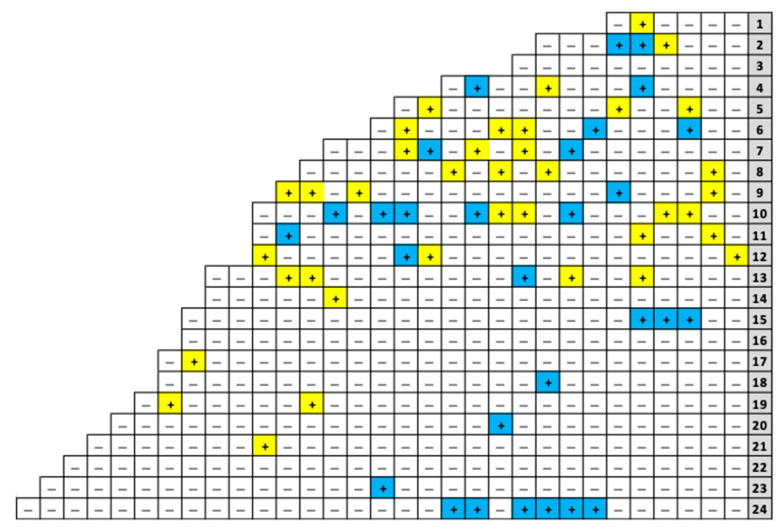
Closeup of rows 1–24 in the northern corner of the ‘Merlot’ vineyard block of interest. To the east of this sub-portion of the ‘Merlot’ block is a riparian area with the Napa River. Each cell represents a single grapevine. Presence or absence of grapevine red blotch virus (GRBV), as determined via visual observations of disease symptoms and diagnostic PCR with specific primers, is denoted with ‘+’ or ‘−’, respectively. Grapevines infected with a GRBV isolate of phylogenetic clade 1 are indicated in blue, and those containing a clade 2 isolate are shown in yellow. A series of ordinary runs analyses indicated aggregations of GRBV-infected vines when all of them were observed contiguously, and in rows 2, 15, and 24 individually. Analysis using ordinary runs of exclusively vines containing GRBV clade 1 isolates indicated aggregations when all infected vines were observed contiguously, and in rows 15 and 24 individually. However, when vines containing GRBV clade 2 isolates were considered exclusively, a random distribution of diseased plants was observed in individual rows and when considered together.

Ordinary runs analysis indicated significant aggregation of diseased vines within the first 24 rows (*Z* = −3.03), particularly in rows 2 (*Z* = −2.04), 15 (*Z* = −2.79), and 24 (*Z* = −3.09) (Figure 3). The remaining symptomatic grapevines (26%, 23/90) in the ‘Merlot’ block mapped to rows 25–90 and occurred randomly with no neighboring, symptomatic vine within the same row or across rows (Figure 2A). Because the disease incidence was low within this area of the vineyard block (0.4%, 23/5227), ordinary runs analysis could not be conducted. Together, spatial analyses indicated a combination of aggregated and randomly distributed, symptomatic vines in the ‘Merlot’ block.

### 3.3. Aggregation of Vines Infected with GRBV Phylogenetic Clade 1 Isolates in the ‘Merlot’ Block

Testing of leaf tissue from symptomatic vines in the ‘Merlot’ block confirmed the presence of GRBV via PCR in all samples (100%, 90/90), as expected. Restriction digests of the PCR products amplifying the replication ORF of the GRBV genome indicated that nearly half of the infected grapevines (43%, 29/67) within the first 24 rows of the ‘Merlot’ block contained a GRBV clade 1 isolate (Figure 3). Ordinary runs analysis showed these vines to be aggregated (*Z* = −4.99), whereas vines infected with clade 2 isolates were not aggregated (*Z* = −1.52), despite making up most GRBV-infected vines (57%, 38/67) in this vineyard area (Figure 3). In the remaining rows of the block (i.e., rows 25–90), most infected grapevines had a GRBV clade 2 isolate (70%, 16/23), and 30% (7/23) of them contained a clade 1 isolate (Figure 2B). These results showed an aggregation of symptomatic grapevines containing GRBV clade 1 isolates in the northern corner of the vineyard, though GRBV clade 2 isolates were present throughout the ‘Merlot’ block and overriding.

### 3.4. Temporal Shift of GRBV Isolates in the ‘Merlot’ Block

**Figure 4:**
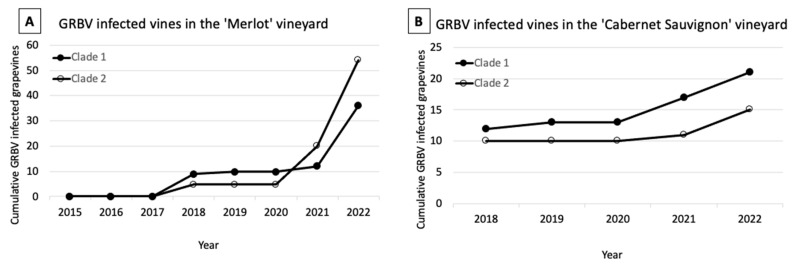
Cumulative counts of newly symptomatic grapevines in the ‘Merlot’ and ‘Cabernet Sauvignon’ blocks of interest based on grapevine red blotch virus (GRBV) phylogenetic clade, as determined via restriction digest of PCR amplicons. (**A**) Newly symptomatic vines in the ‘Merlot’ block in 2015–2019, 2021, and 2022. (**B**) Newly symptomatic vines in the ‘Cabernet Sauvignon’ block in 2018, 2019, 2021, and 2022. No surveys were conducted in 2020 for either block due to travel restrictions related to the COVID-19 pandemic; therefore, counts are carried over from 2019 surveys in each chart. Tissue was collected from each newly symptomatic vine and GRBV presence was confirmed by PCR. Each marker represents cumulative number of infected vines containing GRBV clade 1 or 2 isolates (close and open circles, respectively) found during that year’s surveys.

A temporal analysis of the genetic composition of GRBV isolates in the ‘Merlot’ block revealed a prevalence of clade 1 isolates in 2018 and 2019, while clade 2 isolates were dominant in 2021 and 2022 (Figure 4A). Interestingly, while new infections of grapevines infected with clade 1 isolates were observed each year of the survey, infections with clade 2 isolates persisted at a higher incidence (Supplemental Table S1). This result documented a shift in GRBV isolates over time in infected grapevines of the ‘Merlot’ block with an increased predominance of clade 2 isolates.

### 3.5. GRBV Clade 2 Isolates Surround the ‘Merlot’ Block

Leaf tissue was collected from red blotch symptomatic grapevines in the other ‘Merlot’ block immediately west of the ‘Merlot’ vineyard of interest to identify surrounding GRBV inoculum sources and characterize their genetic composition (Figure 1). All tissue collected tested positive for GRBV (100%, 20/20). In addition, all tissue exclusively contained a GRBV clade 2 isolate, as determined by restriction digesting the replication ORF amplicon obtained via PCR (100%, 20/20 for the other ‘Merlot’ block). Furthermore, the ‘Cabernet franc’ vineyard north of the ‘Merlot’ block was previously found to primarily contain GRBV clade 2 isolates [5,17,36]. 

Similar efforts were made to elucidate the genetic makeup of GRBV isolates in asymptomatic, free-living vines in the proximal riparian area east of the ‘Merlot’ block of interest (Figure 1). Of the five vines tested, four were infected with GRBV (80%, 4/5) via PCR. Those GRBV-infected vines consisted of pure *V. californica* or F1 hybrids of *V. californica* × *V. vinifera* (Table 1). Of those free-living vines containing GRBV, all contained clade 2 isolates (100%, 4/4). 

Together, these results indicated the ‘Merlot’ block of interest was surrounded primarily by grapevines infected with GRBV isolates from phylogenetic clade 2. 

### 3.6. Limited Spread of GRBV in CS169 Vines of the ‘Cabernet Sauvignon’ Vineyard

**Figure 5:**
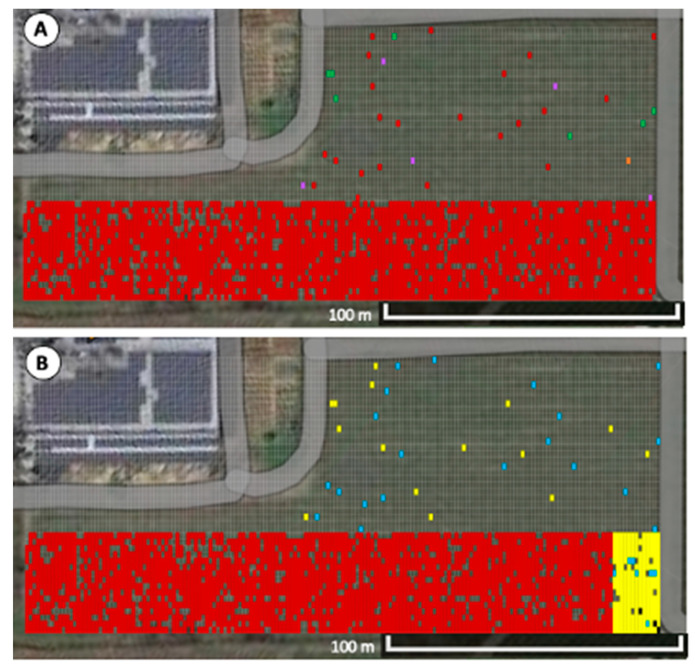
Grapevine red blotch virus (GRBV)-infected vines in a 1.5-hectare ‘Cabernet Sauvignon’ block in 2018–2022. (**A**) Symptomatic CS169 grapevines observed prior to 2018 (red) [36], 2019 (orange), 2021 (purple), and 2022 (green) are indicated. GRBV presence was confirmed in symptomatic vines via PCR. (**B**) Vines infected with a clade 1 isolate are depicted in blue, and those infected with a clade 2 isolate are shown in yellow. Symptomatic CS4 vines for which the genetic makeup of GRBV isolates were characterized are depicted on the bottom right; vines infected with a clade 1 isolate are shown in blue, vines infected with a clade 2 isolate are shown in in yellow, and dead vines in 2022 are shown in black. Adapted with permission from Google Imagery ©2023 NES/Airbus, Maxar Technologies, USDA/FPAC/GEO, Map data ©2023.

A ‘Cabernet Sauvignon’ vineyard was selected for this study to validate observations made in the ‘Merlot’ block. The ‘Cabernet Sauvignon’ block was planted in 2008 with vines of clones CS169 and CS4. Of interest, GRBV incidence was high in CS4 vines which exhibited disease symptoms one year post-planting, while CS169 vines were initially GRBV-negative and asymptomatic, as previously described [36]. Epidemiological surveys for red blotch disease centered on healthy CS169 vines of the ‘Cabernet Sauvignon’ block. A GRBV incidence of 0.6% (17/2799) and 0.9% (24/2799) was reported in 2017 and 2018, respectively, in these vines [36]. One newly symptomatic CS169 vine was found in 2019 (0.9%, 25/2799). Slight increases in disease incidence were observed in CS169 vines in 2021 (1.1%, 30/2799) and 2022 (1.4%, 38/2799) (Figure 5A). 

### 3.7. Isolates of Both GRBV Phylogenetic Clades Are Present in CS169 Vines of the ‘Cabernet Sauvignon’ Vineyard

The presence of GRBV was confirmed in 36 symptomatic CS169 vines tested via PCR in 2022. Two symptomatic vines identified in 2017–2018 had died and could not be tested in 2022. Analyzing the restriction digests of PCR products covering the replication ORF of the GRBV genome, 21 vines contained a clade 1 isolate while 15 contained a clade 2 isolate (Supplemental Table S2). The prevalence of GRBV clade 1 isolates in newly symptomatic CS169 vines remained throughout the surveys (Figure 4B). This work confirms the presence of both GRBV clades in newly infected in CS169 vines in 2017 and 2018 [36], a pattern that persisted after 2019. Taken together, newly symptomatic CS169 vines of the ‘Cabernet Sauvignon’ block had a higher prevalence of clade 1 isolates.

### 3.8. Skewed Distribution of GRBV Clade 1 and Clade 2 Isolates in CS4 Vines of the ‘Cabernet Sauvignon’ Vineyard

Leaf tissue was collected from an aggregated area (*Z =* −1.73) of 189 symptomatic CS4 vines in the ‘Cabernet Sauvignon’ block with a high incidence of GRBV for clade genotyping (Figure 1). All samples tested (100%, 189/189) were identified as positive for GRBV via PCR. Despite being planted at the same time, 81% (153/189) of infected vines contained a GRBV clade 1 isolate and 19% (36/189) contained a clade 2 isolate. These results indicated the presence of GRBV isolates from phylogenetic clades 1 and 2 in diseased CS4 vines of the ‘Cabernet Sauvignon’ block, with a prevalence of GRBV clade 1 isolates.

## 4. Discussion

In this study, distinct red blotch disease epidemiological dynamics were characterized in two nearby vineyards in Napa County, California. The planting material, either the scion or the rootstock, served as the primary source of GRBV inoculum in both vineyards. In addition, secondary spread, likely mediated by *S. festinus*, was documented from either local or background inoculum. In the ‘Merlot’ block, we observed (i) the onset of disease symptoms resulting from GRBV isolates of the minor phylogenetic clade 1 starting three years post-planting, likely because the rootstock served as the primary inoculum source; and (ii) an increased presence of GRBV isolates of the dominant phylogenetic clade 2, likely explained through *S. festinus*-mediated inoculations from background virus sources. More in-depth sequence analysis, such as the genomic diversity fragment, would be required to determine the GRBV isolate identity in background sources in order to elucidate which infections are contributing to secondary spread in the ‘Merlot’ block [16,17]. In the ‘Cabernet Sauvignon’ vineyard, we observed a (i) high incidence of diseased CS4 vines infected with GRBV isolates from both clades and the onset of symptoms one year post planting, likely due to the scion material serving as the primary virus inoculum source; and (ii) limited spatiotemporal increase in proximal diseased CS169 vines infected with clade 1 and 2 isolates, likely via secondary *S. festinus*-mediated inoculations. 

Surveys of the ‘Merlot’ block served as the first case of monitoring red blotch disease onset starting at planting. All previous red blotch epidemiological surveys were performed in diseased vineyards 6–16 years post-planting [5,16,18,36,37]. The ‘Merlot’ vineyard selected for our study was planted in 2015; however, symptoms were not evident until 2018, three years post-planting, particularly within the first 24 rows in which 13% (67/504) of grapevines were symptomatic and aggregated (*Z* = −3.03). The presence of GRBV was confirmed by PCR in all the symptomatic vines and shown to be due to isolates of phylogenetic clades 1 and 2 (Figure 2B). This was particularly interesting as testing of 20 symptomatic vines in the other ‘Merlot’ block and proximal free-living vines (Figure 1) revealed infections of only GRBV clade 2 isolates. Similarly, a neighboring ‘Cabernet franc’ vineyard (Figure 1) contains primarily GRBV clade 2 isolates, as previously reported [5,17,36]. This finding brings into questions how GRBV clade 1 isolates were introduced in the ‘Merlot’ block and further formed a significant aggregation of infected vines in the northern corner (*Z* = −4.99), consisting of 43% of the infected grapevines within the first 24 rows (Figure 3). We hypothesize that this introduction of GRBV isolates from the minor clade 1 was due to infected rootstock material serving as a primary inoculum source. 

Two distinct GRBV epidemiological dynamics were documented in the ‘Merlot’ block. Infections in the first 24 rows contained 74% (67/90) of the symptomatic vines identified throughout the vineyard, while the remainder (26%, 23/90) were distributed within rows 25–90. Disease incidence in these southern rows was extremely low (0.4%, 23/5227) and each symptomatic vine was surrounded by vines neither exhibiting any foliar reddening nor testing positive for GRBV via PCR. While infections throughout the block were initially dominated by GRBV clade 1 isolates, GRBV clade 2 infections became more prevalent in infected vines starting in 2021 (Figure 4A, Supplemental Table S1). This temporal shift of GRBV isolates supports the notion of viruliferous *S. festinus* introducing GRBV clade 2 isolates from outside sources, such as the proximal free-living vines and neighboring vineyard blocks with a high incidence of clade 2 isolates (Figure 1). Furthermore, secondary spread originating from the initial aggregation of infected vines was likely also occurring within this ‘Merlot’ block, as evident by grapevines infected with GRBV clade 1 isolates found in the middle of the block (Figure 2B). 

Differentiating GRBV infections resulting from secondary spread via *S. festinus* and infected rootstock material in the first three to five years post-establishment of a vineyard is very challenging. Our study supports the notion that disease symptoms may not be apparent in vineyards with infected rootstocks until at least three years post-planting. Another study speculated that vines grafted on a GRBV-infected rootstock exhibited disease symptoms four to five years post-planting [38]. Assessing the genetic makeup of GRBV isolates in newly symptomatic vines and diseased vines surrounding the vineyard block of interest, and observing spatiotemporal patterns of symptomatic vines, was crucial in our study to provide insights into the primary source of GRBV inoculum. 

Documenting GRBV in free-living vines supported their role as potential inoculum in secondary spread, confirming previous studies [15,16,17,18,40]. Interestingly, prior accounts of GRBV-infected free-living vines were exclusively reported in *V. californica* hybrids [16,17] or *V. riparia* [18]. Our study is the first to report the presence of GRBV in pure *V. californica*. This result suggests a need to study how *V. californica* might influence the rate of GRBV transmission because a substantially higher *S. festinus*-mediated transmission rate was obtained when a *V. californica* hybrid was used as virus donor or recipient material in comparison with a *V. vinifera* [40].

In the ‘Cabernet Sauvignon’ vineyard of interest, the CS4 vines exhibited red blotch disease symptoms one to two years post-planting [36]. This more rapid onset of disease symptoms compared with the three years observed the ‘Merlot’ block, and the high infection rate of CS4 vines, supports the primary infection source being the scion rather than the rootstock material. GRBV characterization in both CS4 and CS169 plantings were conducted to confirm and expand upon work previously detailed [36]. Interestingly, in a sub portion of the CS4 planting (Figure 1), 81% (153/189) of symptomatic vines were infected with a GRBV clade 1 isolate, and the remaining 19% (36/189) contained a clade 2 isolate (Figure 5B). We hypothesize this mix of GRBV isolates in CS4 vines (Figure 5B) is due to the scion being infected either with clade 1 or clade 2 isolates at planting. Interestingly, no cases of co-infection with clade 1 and clade 2 isolates were identified in infected CS4 vines based on restriction digests of PCR products or sequencing. Despite a very high GRBV incidence in CS4 plantings, very little disease spread was observed in the CS169 plantings within the same ‘Cabernet Sauvignon’ vineyard (Figure 5A). Disease incidence in CS169 vines increased by only 0.5% from 2018 to 2022 (0.9% and 1.4%, respectively), likely due to a ten-fold lower population of *S. festinus* in this block compared to the neighboring ‘Cabernet franc’ block along the riparian area [36,38]. Furthermore, the majority of diseased CS169 vines were infected with GRBV clade 1 isolates (58%, 21/36), while a minority were infected with GRBV clade 2 isolates (42%, 15/36) (Figure 4B, Supplemental Table S2). This distribution of GRBV isolates in initially non-infected CS169 vines reflects a similar isolate distribution to the CS4 vines exhibiting high disease incidence. However, unlike in the ‘Merlot’ block of interest, no shift in GRBV isolates was observed in the ‘Cabernet Sauvignon’ vineyard overtime as GRBV clade 1 isolates remained predominant in the CS4 plantings throughout the surveys (Figure 4B). This result suggests a predominant local spread of GRBV via *S. festinus* in the ‘Cabernet Sauvignon’ vineyard. Furthermore, considering the surrounding inoculum proved key in understanding GRBV epidemiological dynamics in both vineyards. 

Further work is needed to fully characterize red blotch disease onset as it relates to determining the primary GRBV inoculum source as scion or rootstock material. For instance, disease symptoms could be monitored on grafted vines obtained via deliberately assembling different combinations of infected and non-infected rootstock cuttings and scion budwood. This would validate our observation on the latency for disease symptoms in grafted vines in relation to the scion or rootstocks serving as primary GRBV inoculum. Additionally, the distribution of GRBV in rootstocks remains poorly characterized. We hypothesize that, similar to grafted vines, GRBV is most reliably detected in older tissue, such as tissue close to the crown of a rootstock vine [49,50]. Such knowledge is imperative to the development of clean, virus-tested rootstocks that are used to produce the grafted planting material. 

In summary, red blotch disease incidence remained low in a ‘Merlot’ block (1.6%) and CS169 vines of a ‘Cabernet Sauvignon’ block (1.4%) seven and fourteen years post-planting, respectively. Aggregations of diseased vines were observed and explained by the use of infected planting material, whether it be the scion or rootstock. The primary source of virus inoculum had a marked influence on disease onset: one year if the scion was the primary GRBV source or 3–4 years if the rootstock was the primary GRBV source. An aggregation of diseased vines contributed to local *S. festinus*-mediated secondary spread of GRBV in both the ‘Merlot’ and ‘Cabernet Sauvignon’ blocks. In addition, secondary spread from local and background sources was clearly documented in the ‘Merlot’ block. The degree of secondary spread was higher in the ‘Merlot’ block than in the ‘Cabernet Sauvignon’ block, likely due to its proximity to a riparian area with a dense population of GRBV-infected free-living *V. californica* vines, as well as potentially the existence of preferred feeding and/or reproductive plant hosts of *S. festinus.* Our findings stress the need to carefully select planting materials derived from virus-tested vine stocks that supply rootstock cuttings and scion budwood for the production of clean-grafted vines.

## Figures and Tables

**Table 1:** Genotype analysis of free-living grapevines located in a riparian area east of the ‘Merlot’ vineyard of interest as determined using eight SSR markers, and grapevine red blotch virus (GRBV) infection status.

Vine ^a^	Genotype	GRBV Status ^b^
A	F1 hybrid *V. californica* × *V. vinifera* ‘Sauvignon blanc’	+
B	*V. californica*	+
C	F1 hybrid *V. californica* × *V. vinifera* ‘Zinfandel’	+
D	F1 hybrid *V. californica* × *V. vinifera* ‘French Colombard’	+
E	*V. californica*	−

^a^ Sampling sites in the riparian area. Letters correspond to those in Figure 1. ^b^ GRBV was tested via PCR in petiole tissue.

## Data Availability

Raw data will be made available upon request.

## Supplementary Materials

The following supporting information can be downloaded at: https://www.mdpi.com/article/10.3390/v15051184/s1, Table S1: Number of grapevine red blotch virus (GRBV)-infected grapevines in the ‘Merlot’ block of interest based on phylogenetic clade and the year newly symptomatic grapevines were observed; Table S2: Number of grapevine red blotch virus (GRBV) infected CS169 grapevines in the ‘Cabernet Sauvignon’ block of interest based on phylogenetic clade and the year newly symptomatic grapevines were observed.
