# Supplementary material for: Distinct Red Blotch Disease Epidemiological Dynamics in Two Nearby Vineyards
Source: Viruses. 2023 May 17;15(5):1184. doi: 10.3390/v15051184 (PMC10223151; doi:10.3390/v15051184)
Supplement: Supplementary file 1 [file viruses-15-01184-s001.zip › viruses-2406209-supplementary.pdf]

Supplemental Table S1. Number of grapevine red blotch virus (GRBV) infected grapevines in the 'Merlot' block of interest based on phylogenetic clade and the year newly symptomatic grapevines were observed.

Clade[b]	Year[a]								Total[c]
	2015	2016	2017	2018	2019	2020	2021	2022	
Clade 1	0	0	0	9	1	n/a	2	24	36
Clade 2	0	0	0	5	0	n/a	15	34	54
Total[d]	0	0	0	14	1	n/a	17	58	90
Cumulative total[e]	0	0	0	14	15	n/a	32	90	

[a]Year of the survey. [b]GRBV phylogenetic clade determined by restriction digest of PCR amplicons obtained in the replication ORF of the viral genome. [c]Total number of GRBV-infected vines by clade. [d]Total number of GRBV-infected vines by survey year. [e]Cumulative total of GRBV-infected vines observed each year. n/a: not applicable.

Supplemental Table S2. Number of grapevine red blotch virus (GRBV) infected CS169 grapevines in the 'Cabernet Sauvignon' block of interest based on phylogenetic clade and the year newly symptomatic grapevines were observed.

Clade[b]	Year[a]					Total[d]
	2018[c]	2019	2020	2021	2022	
Clade 1	12	1	n/a	4	4	21
Clade 2	10	0	n/a	1	4	15
Total[e]	22	1	n/a	5	8	36
Cumulative total[f]	22	23	n/a	28	36	

[a]Year of the survey. [b]GRBV phylogenetic clade determined by restriction digest of PCR amplicons obtained in the replication ORF of the viral genome. [c]Cumulative results of surveys conducted in 2017 and 2018 in Cieniewicz et al. (2019) and clade analysis (this study). [d]Total number of GRBV-infected vines by clade. [e]Total number of GRBV-infected vines by survey year. [f]Cumulative total of GRBV-infected vines observed each year. n/a: not applicable.
